# Dual-input deep learning system for microbial identification from blood agar plates

**DOI:** 10.1371/journal.pone.0353761

**Published:** 2026-07-27

**Authors:** Takao Naito, Satomi Takei, Shigeki Misawa, Miyuki Kuribara, Yoko Tabe

**Affiliations:** 1 Department of Clinical Microbiology Analysis Development Research, Juntendo University Graduate School of Medicine, Tokyo, Japan; 2 Department of Clinical Laboratory Medicine, Juntendo University Graduate School of Medicine, Tokyo, Japan; 3 Department of Clinical Laboratory Technology, Faculty of Medical Science, Juntendo University, Chiba, Japan; Clinbiocare Technology, INDIA

## Abstract

**Objective:**

The morphological classification of microbial cultures and colonies requires specialized knowledge and experience; therefore, automation in this field remains limited. This study examines the feasibility of automating the identification of pathogenic microbial species from colony images of cultured microorganisms.

**Methods:**

Two distinct image datasets were constructed for 10 clinically relevant species: a colony image dataset, consisting of individual colony images cropped, and a tile image dataset, generated by dividing entire images of the culture plate into tiles. Separate ResNet-50–based models were trained in each dataset, and their outputs were integrated to evaluate the classification performance. Training was conducted using 10,048 colony images and 23,003 tile images collected from 418 strains, and performance was assessed with five-fold cross-validation (K = 5).

**Results:**

The colony image model achieved a sensitivity of 0.934 and a specificity of 0.993, while the tile image model achieved a sensitivity of 0.918 and a specificity of 0.991. Integration of the two models into an ensemble model further improved performance, yielding a sensitivity of 0.955 and a specificity of 0.995 when tested on 76 independent strains.

**Conclusion:**

The ensemble model approach provides high accuracy and robustness, suggesting its potential technical contributions to microbial identification in clinical microbiology laboratories.

## Introduction

Rapid and accurate identification of pathogenic microorganisms is essential for the early diagnosis of infectious diseases and for the selection of appropriate antimicrobial therapy. In particular, the morphological classification of colonies grown on blood agar plates plays a critical role in the initial screening [1]. However, this process requires specialized knowledge and extensive experience, demanding considerable time and effort for training and often resulting in variability in interpretation among clinical technologists [2].

To address this challenge, increasing attention has been focused on artificial intelligence (AI)-based image analysis techniques for the automatic identification of pathogens using colony morphology [3]. Recent advances in deep learning have achieved expert-level performance in pattern recognition and are increasingly being adopted in clinical microbiology laboratories [4]. AI-based studies for microbial image classification have investigated various image types, including Gram-stained smear images obtained directly from clinical specimens [5,6] and colony images derived from culture plates [7,8]. However, most colony-based studies have focused on only a limited number of microbial species or have been conducted under highly controlled conditions that do not adequately reflect the morphological diversity encountered in routine clinical practice [9]. In addition, many existing approaches analyze colonies individually, potentially overlooking the spatial and contextual information distributed across the entire culture plate [7]. These limitations arise largely from the substantial morphological variability among microbial species and culture conditions, which remains a major challenge for accurate classification based solely on visual features [10]. To address these challenges, we adopted a convolutional neural network (CNN)-based Residual Network (ResNet) architecture in this study. CNNs have demonstrated strong performance in image classification tasks [11–14]; however, increasing network depth can impair stable model training because of the vanishing gradient problem during backpropagation, in which gradient signals fail to propagate effectively to earlier layers [15,16]. ResNet overcomes this limitation by introducing shortcut connections that enable residual learning through direct information transfer between layers. This architecture facilitates stable training of deep neural networks and improves classification accuracy compared with non-residual architectures of similar depth [17–21]. Because microbial colony images exhibit substantial morphological variability and complex visual patterns, robust feature extraction using deep architectures is essential for accurate classification. Therefore, we selected ResNet as the backbone architecture for both models in this study. These technological advances may contribute to the standardization and efficiency of microbial diagnostics, particularly in cultured colony classification, where morphological variation is substantial [7].

In this study, we focused on ten clinically relevant species that represent common causative agents of bloodstream infections and frequently encountered pathogens in blood culture diagnostics, including both Gram-positive and Gram-negative organisms. We developed two ResNet-based AI models for colony classification using images obtained from blood agar cultures. One model was trained on a colony image dataset created by extracting individual colony images, and the other model was trained on a tile image dataset generated by systematically dividing entire culture plate images into fixed-size tiles, thereby capturing colony distribution patterns and plate-level contextual features that are not apparent from isolated colony images alone. This dual-input strategy, which combines colony-level morphological features with plate-level spatial and contextual information, represents a distinct approach to clinical colony classification. Previous studies have primarily focused on isolated colony morphology and may therefore overlook broader contextual information distributed across the culture plate. In contrast, our approach integrates both local morphological characteristics and spatial distribution patterns, enabling complementary feature extraction from two different image representations. While colony image analysis captures detailed local morphology, tile-based analysis preserves plate-level contextual information, including colony distribution patterns and spatial relationships that may not be apparent from isolated colony images alone. Each model was trained independently, and their outputs were integrated using an ensemble approach to leverage the complementary strengths of both representations.The classification performance of the system was assessed using standard metrics, while interpretability was evaluated by visualizing high-dimensional feature representations learned by the model using t-distributed stochastic neighbor embedding (t-SNE) [22]. This approach enabled assessment of the degree to which images from each microbial species formed distinct clusters within the feature space.

We then evaluated the classification accuracy, applicability, and interpretability of this approach. Consequently, the ensemble model effectively compensated for the limitations of each individual model and achieved high classification accuracy. This approach is intended as a preliminary screening support tool to assist clinical laboratory staff in the initial assessment of colony identity, rather than as a standalone diagnostic system. By supporting early colony classification, the system has the potential to reduce workload and inter-observer variability inherent in manual inspection.

## Materials and methods

### Construction of the training datasets

Ten clinically relevant species isolated from the clinical microbiology laboratory of the Juntendo University Hospital were selected as target organisms (Data were accessed for research purposes on January 22, 2024). This study was approved by the Institutional Review Board of Juntendo University Hospital (approval number: E23-0101). Among clinically important bacterial species frequently encountered in routine clinical microbiology laboratories, we selected ten species based on their clinical prevalence and their characteristic colony morphologies on blood agar plates. These species were chosen to represent a broad range of morphologic appearances commonly observed in routine laboratory practice. A total of 494 strains were assigned for training (n = 418) and testing (n = 76) (Table 1). To balance the dataset, the number of training images was intentionally increased for species with similar colony morphologies, such as *Escherichia coli* and *Klebsiella pneumoniae*.

**Table 1:** Species and strain distribution in training and test data.

Species	Cultivation time (h)	Number of strains	
		Training set	Test set
*Bacillus subtilis*	16 - 24	31	5
*Campylobacter jejuni*	48 - 96	39	6
*Enterococcus casseliflavus*	24 - 48	15	7
*Escherichia coli*	18 - 24	74	7
*Klebsiella pneumoniae*	18 - 24	80	7
*Moraxella catarrhalis*	24	39	7
*Pseudomonas aeruginosa*	16 - 24	47	7
*Proteus mirabilis*	6 - 17	32	4
*Staphylococcus aureus*	24 - 48	21	13
*Streptococcus pneumoniae*	24 - 48	40	13
Total		418	76

**Fig 1:**
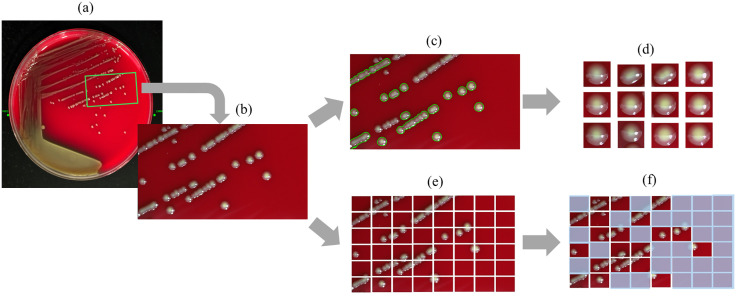
Construction of datasets of colony and tile images. The workflow for extracting colony images and tile images from the whole-plate imaging is shown below. For the magnified view containing a colony from the whole-plate, multiple images (three to five) serve as candidates. Image (a) was acquired with a 50 mm fixed-focus lens, and the others were acquired with a 25 mm fixed-focus lens. (a) Image of the entire culture plate; the green rectangle indicates the magnified region. (b) Magnified view of the selected region. (c) Detected colonies colored green. (d) Cropped colony images used for the colony image dataset. (e) Image of the culture plate divided into 8 × 6 tiles. (f) Extracted tile images, with invalid tiles masked in blue.

Species identification was performed using matrix-assisted laser desorption ionization time of flight mass spectrometry (MALDI-TOF MS) with a MALDI Microflex LT/SH instrument (Bruker Daltonics, Germany) [23,24] and MBT Compass 4.1 software (Bruker Daltonics). The MALDI-TOF MS results, verified by clinical technologists following the CLSI guidelines [25], were used as ground-truth labels. All strains were cultured on trypticase soy agar plates supplemented with 5% sheep blood (Becton, Dickinson Diagnostic Systems, USA) at 35°C. The incubation period ranged from 16 to 96 hours because growth rates differed among species and colonies were intentionally collected at various stages of growth to reflect the morphological variability encountered in routine clinical microbiology laboratories (Table 1). Colony images were acquired using a 4K digital loupe (Shodensha Co., Ltd., Japan) equipped with a 50 mm fixed-focus lens for whole-plate imaging and a 25 mm fixed-focus lens for magnified colony imaging (Fig 1a and 1b). All images were stored in JPEG format at a resolution of 5,440 × 3,060 pixels. No-growth plates were excluded.

Two types of image datasets were constructed from the magnified colony images. The colony image dataset was created by cropping individual colonies (Fig 1c and 1d). Colony contours were detected using the OpenCV module in Python, and geometric features such as circularity and aspect ratio were calculated. Colonies with overlapping contours, strong specular reflections, or indistinct borders with adjacent colonies were excluded. In addition, blurred colonies and those truncated at the edges of the images were manually screened and removed to ensure the quality of the dataset.

The tile image dataset was created by dividing each magnified image into a grid of 8 × 6 tiles (48 segments), each with a resolution of 680 × 510 pixels, as this division produces both horizontally and vertically integer-sized tiles while retaining sufficient spatial information to capture multiple colony morphologies and swarming growth patterns (Fig 1e and 1f). This tile configuration was selected as a practical balance between preserving detailed local morphological features and maintaining broader plate-level spatial and contextual information. Each tile was treated as an independent image and evaluated using edge-strength analysis with OpenCV. Tiles without identifiable objects were excluded, while those above a predefined edge-strength threshold were retained automatically. S1 Fig provides an example using large colonies of *Bacillus subtilis*: when a colony extended across multiple tiles, each corresponding tile was included in the dataset.

Both datasets were generated using the Canny algorithm for edge detection, extracting weak and strong edges with lower and upper thresholds of 40 and 120, respectively. In addition, in the test data designed to reflect reproducibility in clinical laboratory settings, images with low circularity or short edge lengths were excluded, valid colony and tile images were extracted fully automatically. In total, the datasets comprised 10,048 colony images and 23,003 tile images, derived from 418 strains in ten microbial species. The detailed composition of these datasets is summarized in Table 2.

**Table 2:** Composition of the two training datasets.

Species	Number of Strains	Number of images	
		Colony	Tile
*Bacillus subtilis*	31	342	2,750
*Campylobacter jejuni*	39	1,022	2,373
*Enterococcus casseliflavus*	15	1,001	917
*Escherichia coli*	74	935	2,762
*Klebsiella pneumoniae*	80	951	4,687
*Moraxella catarrhalis*	39	1,619	3,105
*Pseudomonas aeruginosa*	32	1,278	1,379
*Proteus mirabilis*	47	773	1,853
*Staphylococcus aureus*	21	783	1,110
*Streptococcus pneumoniae*	40	1,344	2,067
Total	418	10,048	23,003

### Construction of deep learning models

**Fig 2:**
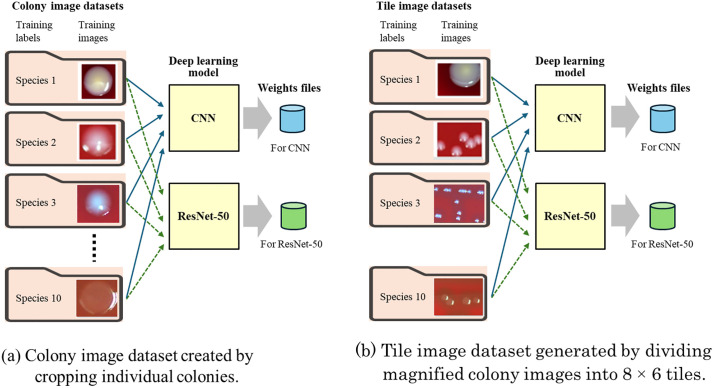
Construction of training models using two image datasets. A flowchart illustrating the generation of weight files for the two types of learning models (CNN and ResNet-50) from the two types of datasets is shown below.

The two types of input datasets, the colony image dataset and the tile image dataset, were used to train and evaluate CNN and Residual Networks (ResNets) (Fig 2). Each training image was assigned a true label corresponding to its microbial species, as determined in the laboratory using MALDI-TOF MS.

During training, the prediction error (loss) between the model outputs and the true labels was calculated using the Cross-Entropy Loss function [26]. The gradients of this loss were propagated backward to update the network weights, thus minimizing the error, as previously described [27]. ResNet architectures range in depth from 18 to 152 layers; in this study, we adopted a CNN four-layer and ResNet-50 with 50 layers as the primary models. Both architectures were trained on the colony and tile datasets. The training was not based on transfer learning. The model was trained from scratch using the dataset generated in this study. Model development and training were conducted using the Python PyTorch library on a GPU workstation equipped with an NVIDIA GeForce RTX 3060.

### Evaluation of the model using training data

The optimal number of training epochs for CNN and ResNet-50 models was determined based on the classification accuracy (i.e., the rate of matching between the predicted and true labels) and the loss values. Training was stopped when the learning curves converged and both accuracy and loss plateaued, indicating model convergence. At that point, the models were saved as  .pth files. During training, the weights of the internal nodes in the neural networks were iteratively updated to capture the feature representations learned from the training data.

To quantitatively assess model performance, K-fold cross-validation (K = 5) was used and confusion matrices were generated. In addition, sensitivity, specificity, precision and F1 scores were calculated to validate the effectiveness of the models.

The sensitivity was calculated as true positive / (true positive + false negative), the specificity was true negative / (true negative + false positive), the precision was true positive / (true positive + false positive) and the F1 score was 2 × sensitivity × precision / (sensitivity + precision).

### Evaluation using test data

**Fig 3:**
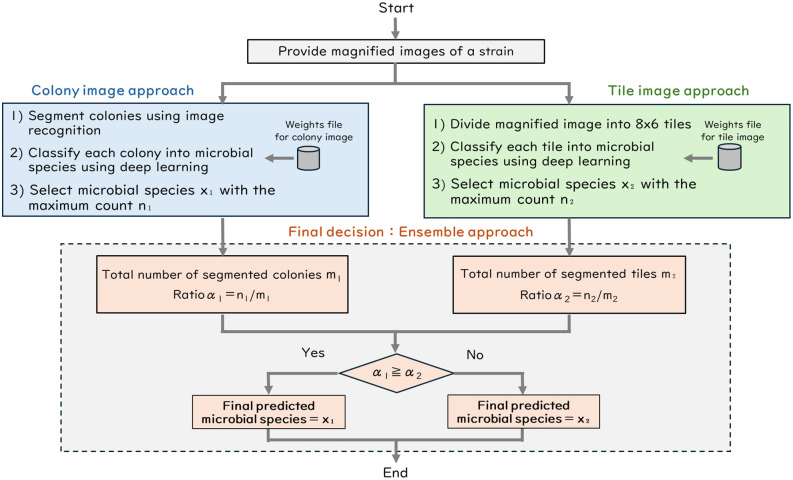
Workflow for prediction of microbial species using the ensemble model approach. Magnified images of each strain were processed using two complementary approaches. In the colony image approach (left), individual colonies were segmented, classified into microbial species using deep learning, and the species with the highest count (x_1_) was selected. In the tile image approach (right), each magnified image was divided into 8 × 6 tiles, classified into microbial species, and the species with the highest count (x_2_) was selected. For the final prediction, the ratios α_1_ (n_1_/m_1_) and α_2_ (n_2_/m_2_) were calculated, where n indicates the maximum count and m the total number of colonies or tiles. When α_1_ ≥ α_2_, the final predicted species was x_1_; otherwise, x_2_ was chosen.

To evaluate the performance of the trained CNN and ResNet-50 models, we prepared an independent test data of 76 strains cultured at 35°C for 6–96 hours not included in the training set (Table 1). The evaluation workflow for the test data is illustrated in Fig 3. Colony and tile images were automatically extracted and cleaned from test data using the same workflow as for the training dataset. In the evaluation, both the colony image model (as a colony level analysis) and the tile image model (as a tile level analysis) were applied to classify each extracted colony image into one of the ten target species. For each strain, the predicted species labels were tallied separately for the two models and the most frequently predicted species was selected as the representative prediction, denoted as x₁ for the colony image model and x_2_ for the tile image model.

To determine the final species prediction, the outputs of the two models were integrated using an ensemble model approach based on the relative ratios of the species counts predicted by the two models. When both models predicted the same species, that species was adopted as the final result. In cases where the predictions differed, the species associated with the highest overall prediction count or score among the two models was selected as the final outcome.

#### Model visualization.

To assess the validity of species classification by the ResNet-50 deep learning model, t-distributed Stochastic Neighbor Embedding (t-SNE) was used to reduce high-dimensional feature vectors to a two-dimensional space. This reduction in dimensionality enabled visualization of clustering patterns that reflect classification similarity [22,28]. The analysis was performed separately for the colony image model and the tile image model to evaluate their respective classification behaviors. To visualize the feature distributions extracted from the trained ResNet-50, we applied t-SNE using the implementation provided in the Python module scikit-learn (version 1.3.0). The features were reduced to two dimensions using t-SNE with the following hyperparameters: the number of components was set to 2 and the perplexity, which determines cluster density, was set to 20. All other parameters were kept at their default values.

## Results

### Construction and evaluation of deep learning models

**Fig 4:**
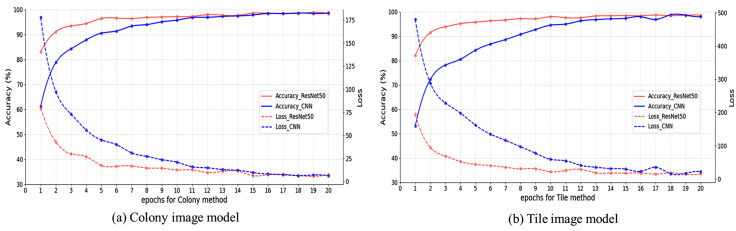
Training performance of CNN and ResNet-50 models using colony and tile image datasets. Accuracy and loss curves are shown for both models a and b. The optimal number of training epochs was determined from the point at which the curves stabilized, indicating the convergence of the model.

During model training, both the CNN and ResNet-50 architectures showed rapid convergence. In 5-fold cross-validation, classification accuracy and loss values stabilized within approximately 20 epochs for both the colony and tile image models, indicating sufficient learning without overfitting. Consequently, final training was conducted for 20 epochs using the entire dataset. Both CNN and ResNet-50 models achieved classification accuracies that exceeded 98% on the colony and tile image datasets (Fig 4). The loss values converged within the range of 2.18–7.70 for the colony image model and 13.9–15.7 for the tile image model.

To further evaluate the performance of the model, we generated confusion matrices (S2 Fig) and calculated classification metrics using 5-fold cross-validation (S1 and S2 Tables). The CNN model achieved a sensitivity of 0.797 and an F1 score of 0.791 for the tile image dataset, indicating relatively low performance. In contrast, the ResNet-50 model achieved higher scores in all metrics, with a sensitivity of 0.934 and an F1 score of 0.932 for the colony image dataset, and a sensitivity of 0.918 and an F1 score of 0.917 for the tile image dataset. Based on these results, the ResNet-50 model was selected for all subsequent analyses.

### Evaluation using the test data

The performance of the ResNet-50 identification system was assessed using a test dataset comprising 76 strains of ten microbial species not included in the training (Table 1). Following the evaluation workflow (Fig 3), three models were compared: the colony image model, the tile image model, and the integrated ensemble model. The colony and tile models achieved correct identification rates of 92.0% (69/75 strains; one strain was excluded because no valid colony image could be extracted) and 90.8% (69/76 strains), respectively, whereas the ensemble model attained 96.1% (73/76 strains) in Table 3, demonstrating the highest accuracy. Confusion matrices were generated (S3 Fig). Species-level performance metrics were averaged across 10 classes and summarized in Table 4. The ensemble model yielded the most favorable results, with sensitivity of 0.955, specificity of 0.995, precision of 0.966, and an F1 score of 0.959. Collectively, these findings demonstrate that integration of the colony and tile models enhances robustness and provides superior classification performance compared with either model alone.

**Table 3:** Identification performance of three models by CNN and ResNet-50.

Model	Accuracy (%)	
	CNN	ResNet-50
Colony image	74.7	92.0
Tile image	72.3	90.8
Ensemble	77.6	96.1

**Table 4:** Performance metrics of ResNet-50 models (colony image model, tile image model, and ensemble model) evaluated on the test data.

Species	Colony image model				Tile image model				Ensemble -model			
	Sensitivity	Specificity	Precision	F1 score	Sensitivity	Specificity	Precision	F1 score	Sensitivity	Specificity	Precision	F1 score
*Bacillus subtilis*	1.000	1.000	1.000	1.000	1.000	1.000	1.000	1.000	1.000	1.000	1.000	1.000
*Campylobacter jejuni*	0.833	1.000	1.000	0.909	1.000	1.000	1.000	1.000	0.833	1.000	1.000	0.909
*Enterococcus casseliflavus*	1.000	0.984	0.875	0.933	0.857	1.000	1.000	0.923	0.857	0.985	0.857	0.857
*Escherichia coli*	0.857	1.000	1.000	0.923	0.857	1.000	1.000	0.923	0.857	1.000	1.000	0.923
*Klebsiella pneumoniae*	0.571	0.985	0.800	0.667	1.000	0.954	0.700	0.824	1.000	0.985	0.875	0.933
*Moraxella catarrhalis*	1.000	1.000	1.000	1.000	1.000	1.000	1.000	1.000	1.000	1.000	1.000	1.000
*Pseudomonas aeruginosa*	1.000	0.984	0.875	0.933	1.000	1.000	1.000	1.000	1.000	1.000	1.000	1.000
*Proteus mirabilis*	0.750	1.000	1.000	0.857	0.250	1.000	1.000	0.400	1.000	1.000	1.000	1.000
*Staphylococcus aureus*	1.000	0.949	0.813	0.897	0.846	1.000	1.000	0.917	1.000	1.000	1.000	1.000
*Streptococcus pneumoniae*	1.000	1.000	1.000	1.000	1.000	0.933	0.765	0.867	1.000	0.984	0.929	0.963
Average	0.901	0.990	0.936	0.912	0.881	0.989	0.946	0.885	0.955	0.995	0.966	0.959

The colony image model is a colony level analysis and the tile image model is a tile level analysis. The ensemble model is based on majority voting of the species counts predicted by the two models.

### Visualization of classification clusters

**Fig 5:**
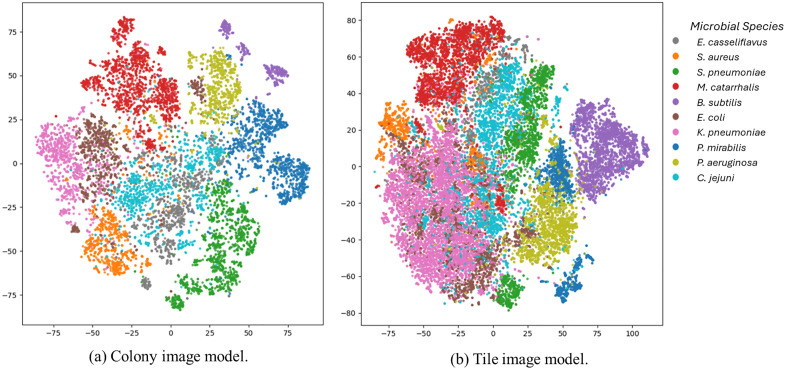
Visualization of species similarity using training data with t-SNE. Each of the ten species is colored individually. Despite some overlapping among species with strong morphological similarities, the data points are broadly separated as clusters. (a) Colony image model trained on 10,048 images. (b) Tile image model trained on 23,003 images.

The high-dimensional features extracted from the colony and tile image models were reduced to two dimensions using t-distributed stochastic neighbor embedding (t-SNE) [27] and visualized in Fig 5.

The 2D t-SNE plots revealed that the clusters representing *E. coli* (brown) and *K. pneumoniae* (pink), as well as *Enterococcus casseliflavus* (gray) and *Campylobacter jejuni* (light blue), were closely positioned and partially overlapped in both models. These patterns are consistent with the known morphological similarities of these species and suggest that the models captured shared feature representations (S4 and S5 Fig., respectively).

Notable differences in clustering behavior were also observed between the two models. For example, the cluster corresponding to *Streptococcus pneumoniae* (green) formed a single and well-defined region in the colony image model, while it appeared as two elongated clusters in the tile image model. This distribution is consistent with the superior precision and F1 score achieved by the colony image model for *S. pneumoniae* (Table 4). In other words, colonies of *S. pneumoniae* share similar morphological features due to their spherical shape; however, tile images may exhibit greater variability in feature distribution, reflecting a wider diversity of local appearance patterns (S6 Fig). Similarly, *Proteus mirabilis* (blue) and *Pseudomonas aeruginosa* (light green) were well separated in the colony image model but showed blurred boundaries in the tile image model. In contrast, *B. subtilis* (purple), which typically forms spreading colonies, appeared as multiple small, scattered clusters in the colony image model but formed a single cohesive cluster in the tile image model.

Together, these findings demonstrate that colony and tile image models capture distinct yet complementary aspects of species-specific morphological features, reinforcing the value of their integration in microbial classification. Even when the perplexity, a parameter of t-SNE related to cluster density, was varied between 10 and 50, the overall clustering patterns showed little difference.

## Discussion

In this study, we developed a dual deep learning classification system that integrates two types of image datasets, colony images and tile images. The ResNet-50-based ensemble model achieved a species-level identification accuracy of 96.1% for microbial colonies cultured on blood agar plates. The Transformer-based Cascade R-CNN model has been reported to achieve an accuracy of 76.7% [7]. It was applied to five species in the AGAR (Annotated Germs for Automated Recognition) dataset (*Staphylococcus aureus*, *B. subtilis*, *P. aeruginosa*, *E. coli*, and *Candida albicans*). Although the AGAR model and our ensemble model cannot be directly compared due to differences in experimental conditions, we analyzed their differences as outlined below. The first difference concerns image acquisition conditions. In this study, all images were obtained under rigorously standardized and fixed conditions using the same equipment, lighting, and resolution settings. In contrast, the AGAR dataset comprises heterogeneous images acquired under diverse imaging conditions. The second difference is the introduction of an ensemble model integrating a colony image model and a tile image model.

The colony image dataset yielded higher classification performance than the tile image dataset, which may be attributed to the higher information density of individually extracted colony images, where morphologically relevant features are consistently centered within each image. In contrast, tile images generated by mechanical division of whole-plate photographs inevitably include tiles lacking colonies or containing only partial colony representations, potentially introducing noise during training. The integration of both models into an ensemble therefore leverages the respective strengths of each approach, ultimately achieving higher overall classification accuracy than either model alone.

With the ensemble model, limitations of the colony image model alone became less pronounced for species exhibiting characteristic growth patterns. For example, in *P. mirabilis*, which shows a spreading growth morphology, the colony image model had difficulty capturing the overall colony structure, whereas the tile image model was able to effectively extract and classify relevant features. Similarly, for *B. subtilis*, which forms large and expansive colonies, more consistent clustering was observed in the tile image model, indicating its effectiveness in capturing spatial morphological characteristics.

The t-SNE analysis [22] visually demonstrated the complementary nature of the two approaches. The colony image model exhibited clearer cluster separation than the tile image model, which was consistent with its higher sensitivity and precision (S2 Table). For example, *P. mirabilis* and *P. aeruginosa* were well separated in the colony image model, whereas their boundaries were less distinct in the tile image model. In contrast, morphologically similar species, such as *C. jejuni* and *E. casseliflavus*, showed overlapping clusters in the colony image model, corresponding to the misclassification patterns observed in the confusion matrices (S2 Fig). Importantly, no dependency on perplexity, a parameter related to local cluster density in t-SNE, was observed across the tested range, indicating that the visualization results were relatively stable and that the interpretation should not rely on specific t-SNE settings. Rather than attributing performance differences to t-SNE-derived clustering patterns, these findings suggest that the two models capture distinct and complementary features. By integrating the strengths of both models, the ensemble model achieved higher accuracy than either model alone. This integration mitigated the limitations inherent to each individual approach and improved overall robustness.

This study has several limitations. First, the morphology of the microbial colony varies by strain and growth phase. Although images were acquired at optimized time points, a broader clinical application will require datasets that include diverse culture conditions and agar types. Second, the standardization of imaging protocols — including equipment, lighting, and acquisition angle — remains essential for reproducibility. Establishing practical guidelines for image acquisition will be an important step toward reducing variability. Third, the dataset was limited to ten species. Future research should expand coverage to additional clinically relevant species, explore more advanced feature extraction techniques, and assess newer or deeper network architectures. Finally, the present study was designed to evaluate the feasibility of applying AI for microbial identification, while intentionally excluding factors of complexity. Therefore, the analysis was limited to ten clinically relevant microbial species, and neither polymicrobial cultures nor artifacts caused by residual materials from the primary culture medium were considered. In future work, we plan to expand this study by incorporating more complex conditions.

In conclusion, the finding that the ensemble model outperforms either individual model provides stronger and more direct evidence that the colony- and tile-based predictions offer complementary information. With expanded datasets and standardized imaging protocols, this approach has the potential to provide technical contributions to microbial identification in clinical microbiology laboratories.

## Conclusion

We developed a dual-input deep learning system for microbial identification from blood agar plates using an ensemble of colony- and tile-based image models. The system achieved high accuracy and robustness and may serve as a useful preliminary screening support tool for microbial identification in clinical microbiology laboratories.

## Supporting information

S1 Fig Tile images derived from large microbial colonies. (a) Whole culture plate image and the green rectangle area is target of magnification. (b) Magnified view of the green rectangle area. (c) Segmented and extracted tile images with invalid tiles masked in blue.
(TIF)

S2 Fig Confusion matrices obtained by fivefold cross-validation (K = 5) on training dataset. (a) Colony image model with CNN trained on 10,048 images. (b) Tile image model with CNN trained on 23,003 images. (c) Colony image model with ResNet-50 trained on 10,048 images. (d) Tile image model with ResNet-50 trained on 23,003 images. The x axis represents the true labels, and the y axis represents the predicted labels. The color intensity in the heatmaps indicates the number of correct and incorrect predictions, with darker blue corresponding to correct classifications and lighter blue to misclassifications.
(PNG)

S3 Fig Confusion matrices obtained by fivefold cross-validation (K = 5) on test dataset. (a) Colony image model with ResNet-50 tested on 76 images. (b) Tile image model with ResNet-50 tested on 76 images (c) Ensemble model with ResNet-50 tested on 76 images. The x axis represents the true labels, and the y axis represents the predicted labels. The color intensity in the heatmaps indicates the number of correct and incorrect predictions, with darker blue corresponding to correct classifications and lighter blue to misclassifications.
(PNG)

S4 Fig Morphological similarity between *E. col* and *K. pneumoniae.* Both are spherical in shape, making them difficult to distinguish by visual inspection.
(TIF)

S5 Fig Morphological similarity between *E. casseliflavus* and *C. jejuni.* Depending on the culture conditions, *C. jejuni* may become spherical and closely resemble *E. casseliflavus*.
(TIF)

S6 Fig Colony and tile images of *S. pneumoniae.* a. Colony image. b. Tile image.
(PNG)

S1 Table Results of CNN cross validation (k = 5). (DOCX)

S2 Table Results of ResNet-50 cross validation (k = 5). (DOCX)
