# Supplementary figures and images for: Dual-input deep learning system for microbial identification from blood agar plates
Source: PLoS One. 2026 Jul 27;21(7):e0353761. doi: 10.1371/journal.pone.0353761 (PMC13405119; doi:10.1371/journal.pone.0353761)

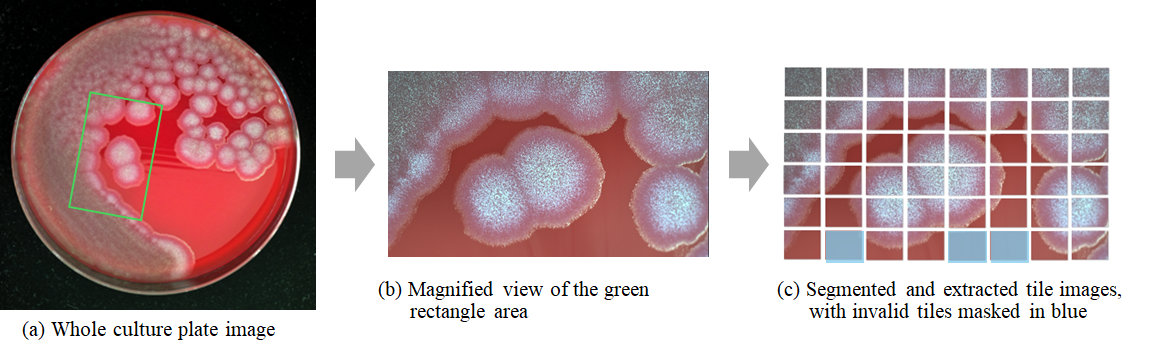

Supplement: S1 Fig — (a) Whole culture plate image and the green rectangle area is target of magnification. (b) Magnified view of the green rectangle area. (c) Segmented and extracted tile images with invalid tiles masked in blue. (TIF) [file pone.0353761.s001.tif]

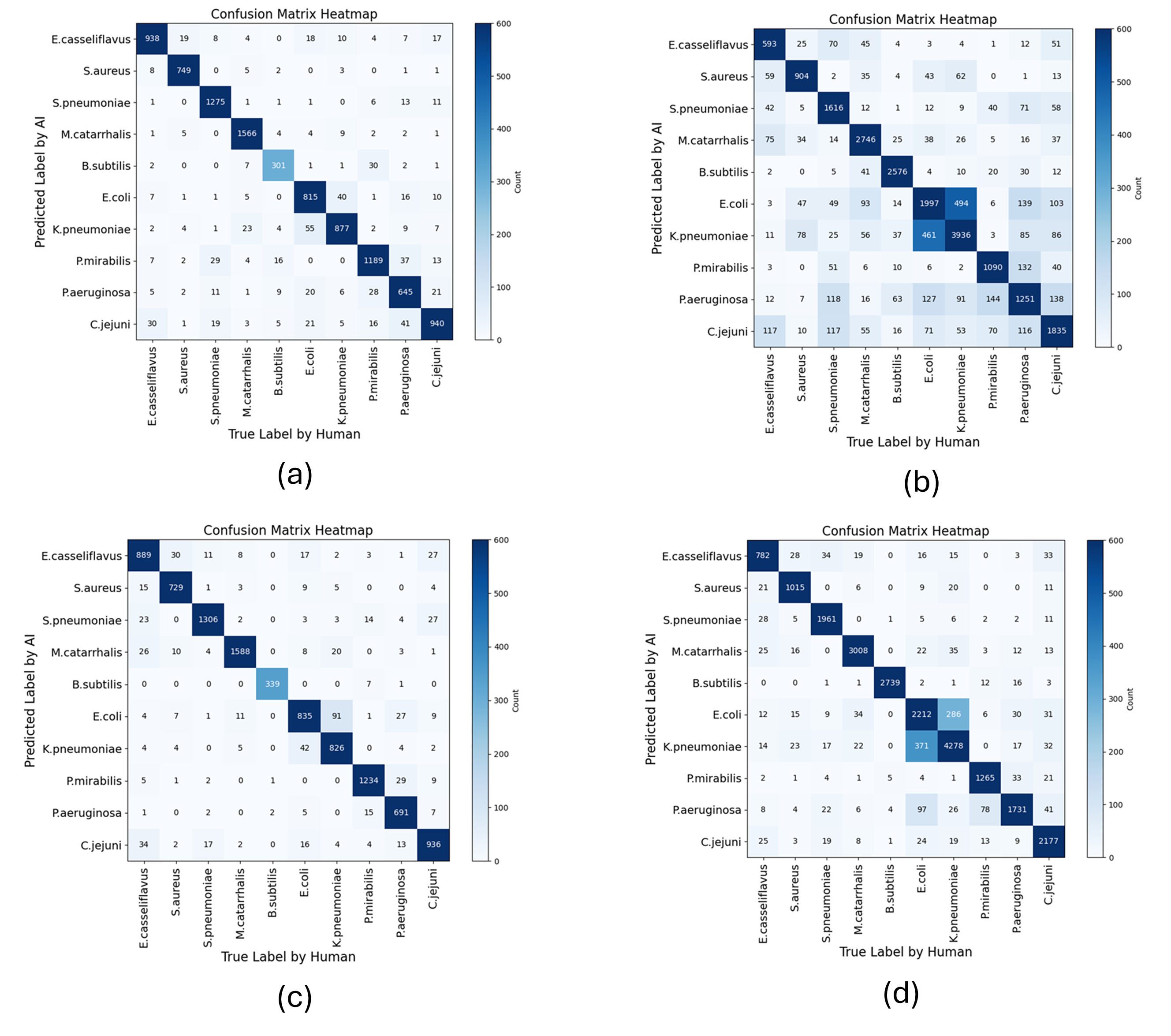

Supplement: S2 Fig — (a) Colony image model with CNN trained on 10,048 images. (b) Tile image model with CNN trained on 23,003 images. (c) Colony image model with ResNet-50 trained on 10,048 images. (d) Tile image model with ResNet-50 trained on 23,003 images. The x axis represents the true labels, and the y axis represents the predicted labels. The color intensity in the heatmaps indicates the number of correct and incorrect predictions, with darker blue corresponding to correct classifications and lighter blue to misclassifications. (PNG) [file pone.0353761.s002.png]

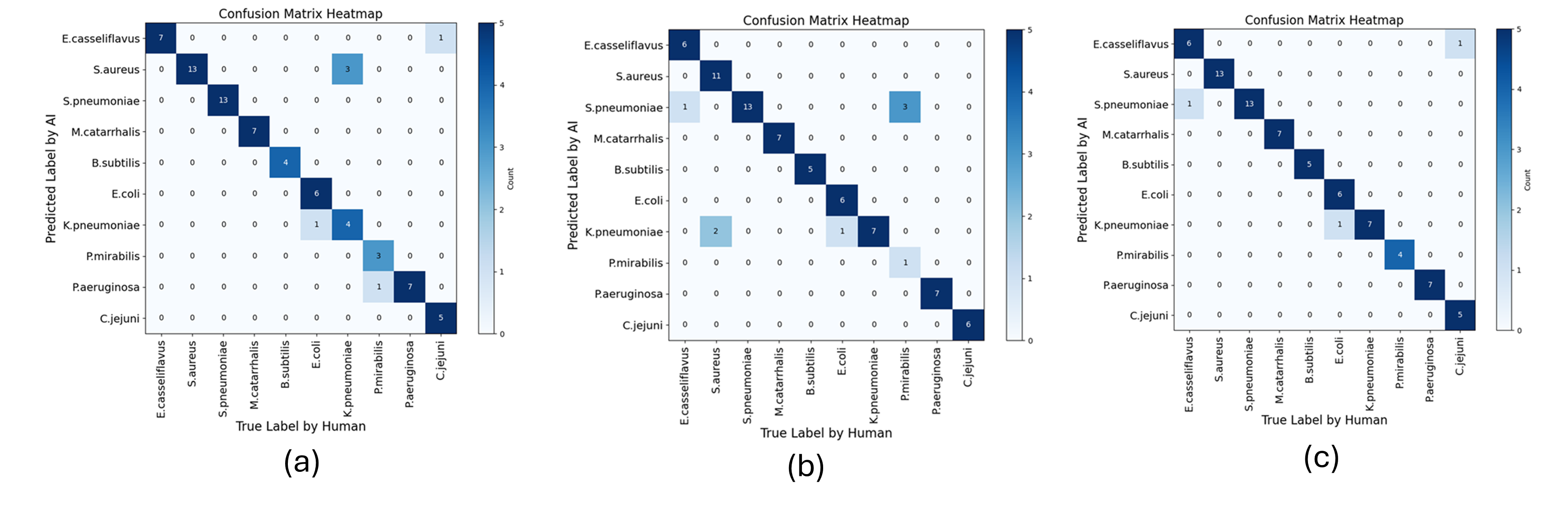

Supplement: S3 Fig — (a) Colony image model with ResNet-50 tested on 76 images. (b) Tile image model with ResNet-50 tested on 76 images (c) Ensemble model with ResNet-50 tested on 76 images. The x axis represents the true labels, and the y axis represents the predicted labels. The color intensity in the heatmaps indicates the number of correct and incorrect predictions, with darker blue corresponding to correct classifications and lighter blue to misclassifications. (PNG) [file pone.0353761.s003.png]

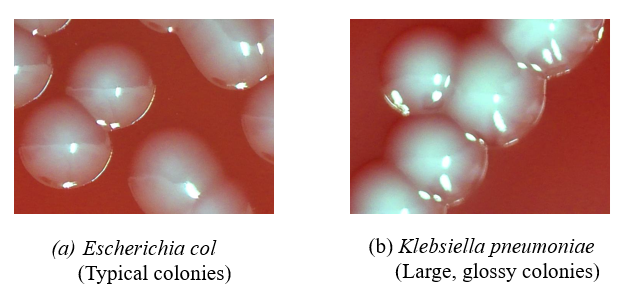

Supplement: S4 Fig — Both are spherical in shape, making them difficult to distinguish by visual inspection. (TIF) [file pone.0353761.s004.tif]

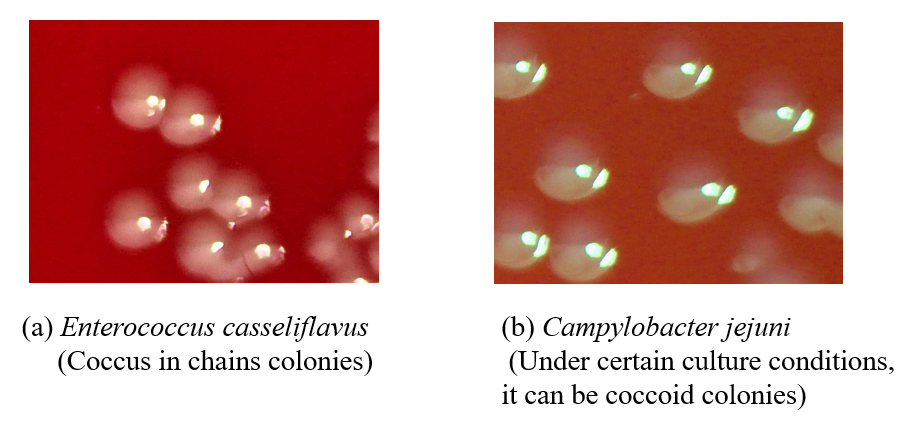

Supplement: S5 Fig — Depending on the culture conditions, C. jejuni may become spherical and closely resemble E. casseliflavus. (TIF) [file pone.0353761.s005.tif]

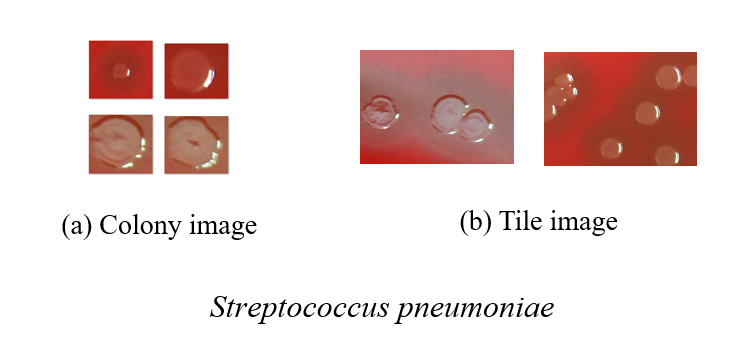

Supplement: S6 Fig — a. Colony image. b. Tile image. (PNG) [file pone.0353761.s006.png]
