# Supplementary material for: Dual-input deep learning system for microbial identification from blood agar plates
Source: PLoS One. 2026 Jul 27;21(7):e0353761. doi: 10.1371/journal.pone.0353761 (PMC13405119; doi:10.1371/journal.pone.0353761)
Supplement: S1 Table — (DOCX) [file pone.0353761.s007.docx]

**S1 Table.** Results of CNN cross validation (k = 5)

| Species |  | Colony image model | | | |  | Tile image model | | | |
| --- | --- | --- | --- | --- | --- | --- | --- | --- | --- | --- |
|  |  | Sensitivity | Specificity | Precision | F1 score |  | Sensitivity | Specificity | Precision | F1 score |
| *Bacillus subtilis* |  | 0.872 | 0.996 | 0.880 | 0.876 |  | 0.954 | 0.991 | 0.937 | 0.945 |
| *Campylobacter jejuni* |  | 0.870 | 0.991 | 0.920 | 0.894 |  | 0.746 | 0.974 | 0.773 | 0.759 |
| *Enterococcus casseliflavus* |  | 0.915 | 0.993 | 0.937 | 0.926 |  | 0.734 | 0.985 | 0.647 | 0.688 |
| *Escherichia coli* |  | 0.910 | 0.987 | 0.872 | 0.890 |  | 0.678 | 0.962 | 0.723 | 0.700 |
| *Klebsiella pneumoniae* |  | 0.891 | 0.992 | 0.922 | 0.906 |  | 0.824 | 0.959 | 0.840 | 0.832 |
| *Moraxella catarrhalis* |  | 0.982 | 0.994 | 0.967 | 0.975 |  | 0.910 | 0.982 | 0.884 | 0.897 |
| *Pseudomonas aeruginosa* |  | 0.862 | 0.986 | 0.834 | 0.848 |  | 0.636 | 0.971 | 0.675 | 0.655 |
| *Proteus mirabilis* |  | 0.917 | 0.990 | 0.930 | 0.923 |  | 0.813 | 0.987 | 0.790 | 0.802 |
| *Staphylococcus aureus* |  | 0.974 | 0.996 | 0.957 | 0.965 |  | 0.805 | 0.991 | 0.814 | 0.810 |
| *Streptococcus pneumoniae* |  | 0.974 | 0.992 | 0.949 | 0.961 |  | 0.866 | 0.979 | 0.782 | 0.822 |
| Average |  | 0.917 | 0.992 | 0.917 | 0.917 |  | 0.797 | 0.978 | 0.787 | 0.791 |
