# Supplementary material for: Dual-input deep learning system for microbial identification from blood agar plates
Source: PLoS One. 2026 Jul 27;21(7):e0353761. doi: 10.1371/journal.pone.0353761 (PMC13405119; doi:10.1371/journal.pone.0353761)
Supplement: S2 Table — (DOCX) [file pone.0353761.s008.docx]

**S2 Table.** Results of ResNet-50 cross validation (k = 5)

| Species |  | Colony image model | | | |  | Tile image model | | | |
| --- | --- | --- | --- | --- | --- | --- | --- | --- | --- | --- |
|  |  | Sensitivity | Specificity | Precision | F1 score |  | Sensitivity | Specificity | Precision | F1 score |
| *Bacillus subtilis* |  | 0.977 | 1.000 | 0.991 | 0.984 |  | 0.987 | 0.999 | 0.996 | 0.991 |
| *Campylobacter jejuni* |  | 0.911 | 0.990 | 0.916 | 0.913 |  | 0.947 | 0.991 | 0.917 | 0.932 |
| *Enterococcus casseliflavus* |  | 0.900 | 0.988 | 0.888 | 0.894 |  | 0.841 | 0.994 | 0.853 | 0.847 |
| *Escherichia coli* |  | 0.847 | 0.989 | 0.893 | 0.869 |  | 0.839 | 0.973 | 0.801 | 0.820 |
| *Klebsiella pneumoniae* |  | 0.931 | 0.986 | 0.869 | 0.899 |  | 0.896 | 0.978 | 0.913 | 0.904 |
| *Moraxella catarrhalis* |  | 0.957 | 0.996 | 0.981 | 0.969 |  | 0.960 | 0.995 | 0.969 | 0.964 |
| *Pseudomonas aeruginosa* |  | 0.956 | 0.991 | 0.894 | 0.924 |  | 0.858 | 0.994 | 0.934 | 0.895 |
| *Proteus mirabilis* |  | 0.963 | 0.995 | 0.966 | 0.964 |  | 0.946 | 0.995 | 0.917 | 0.932 |
| *Staphylococcus aureus* |  | 0.952 | 0.994 | 0.931 | 0.941 |  | 0.938 | 0.996 | 0.914 | 0.926 |
| *Streptococcus pneumoniae* |  | 0.945 | 0.996 | 0.972 | 0.958 |  | 0.970 | 0.995 | 0.949 | 0.959 |
| Average |  | 0.934 | 0.993 | 0.930 | 0.932 |  | 0.918 | 0.991 | 0.916 | 0.917 |
